# Land-use transitions and carbon emissions of aquaculture expansion in the Brazilian Amazon

**DOI:** 10.1126/sciadv.aec5472

**Published:** 2026-08-14

**Authors:** Felipe S. Pacheco, Sebastian A. Heilpern, Rafael M. Almeida, Suresh A. Sethi, Marcela Miranda, Nicholas Ray, Laura Greenstreet, Joshua Fan, Brendan H. Rappazzo, Anmol Kabra, Marta E. Ummus, Ícaro B. Alves, Nathan O. Barros, Jucilene Cavali, Gabriel M. Cesar, Carolina R. Doria, Kathryn J. Fiorella, Bruce R. Forsberg, Marcelo Gomes da Silva, Daniel A. Maciel, David McGrath, Peter B. McIntyre, Patricia Moraes-Valenti, Ilce Oliveira, Jean P. H. B. Ometto, Fabio Roland, Adry Trindade, Wagner C. Valenti, Aline M. Valerio, Xiangtao Xu, Carla P. Gomes, Alexander S. Flecker

**Affiliations:** ^1^ Department of Ecology and Evolutionary Biology, Cornell University, Ithaca, NY, USA.; ^2^ Doerr School of Sustainability, Stanford University, Stanford, CA, USA.; ^3^ O’Neill School of Public and Environmental Affairs, Indiana University, Bloomington, IN, USA.; ^4^ Aquatic Research and Environmental Assessment Center, Brooklyn College, City University of New York, New York, NY, USA.; ^5^ Impacts, Adaptation and Vulnerability Division, National Institute for Space Research, São José dos Campos, São Paulo, Brazil.; ^6^ School of Marine Science and Policy, University of Delaware, Lewes, DE, USA.; ^7^ Department of Computer Science, Cornell University, Ithaca, NY, USA.; ^8^ Embrapa Fisheries and Aquaculture, Palmas, Tocantins, Brazil.; ^9^ Department of Biology, Federal University of Juiz de Fora, Juiz de Fora, Minas Gerais, Brazil.; ^10^ Department of Animal Science, Federal University of Rondônia, Porto Velho, Rondônia, Brazil.; ^11^ National Institute for Amazonian Research, Manaus, Amazonas, Brazil.; ^12^ School of Earth, Environmental, and Marine Sciences, University of Texas Rio Grande Valley, Edinburg, TX, USA.; ^13^ Earth Innovation Institute, San Francisco, CA, USA.; ^14^ Aquaculture Center, São Paulo State University, Jaboticabal, São Paulo, Brazil.

## Abstract

Accounting for land-use change underlying food sector expansion is critical for conserving ecosystem integrity. This challenge is particularly acute in the Amazon, where the conversion of forests for food production is pronounced and has broad implications for global biodiversity and the cycling of water and carbon. Here, we analyze land-use transitions associated with aquaculture development in the Brazilian Amazon. We found that a 450-fold increase in aquaculture pond area from 1985 to 2022 occurred primarily on pasturelands (87%) but also included wetland conversion (4%) and deforestation (5%). Notably, continued growth can be sustained within already altered landscapes: In Rondônia, the leading aquaculture state in the Amazon, production could double by converting only 1.2% of the total degraded pasture area. While directing aquaculture expansion toward degraded pastures could reduce further natural habitat conversion and carbon costs historically associated with food production in the Amazon, realizing this potential will depend on strong governance and enforceable land-use policies to guide siting decisions.

## INTRODUCTION

The conversion of natural habitats to produce food is a leading driver of global biodiversity loss (*1*) and climate change (*2*). Inland aquaculture is expanding rapidly with the promise of producing food with a lower land footprint than other forms of animal-sourced food production (e.g., cattle) (*3*). However, the environmental footprinting of aquaculture typically encompasses only the present area of land under production and land needed to produce inputs (e.g., feed) (*4*), ignoring historical land-use change dynamics associated with the sector’s expansion. This poor accounting could under- or overestimate the impact of aquaculture on biodiversity and climate, especially in regions with large tracts of standing forests such as the Amazon (*5*). Accurately assessing land-use dynamics associated with aquaculture expansion is key to understanding its ecosystem impacts and identifying strategies for more sustainable food production.

**Fig. 1.**
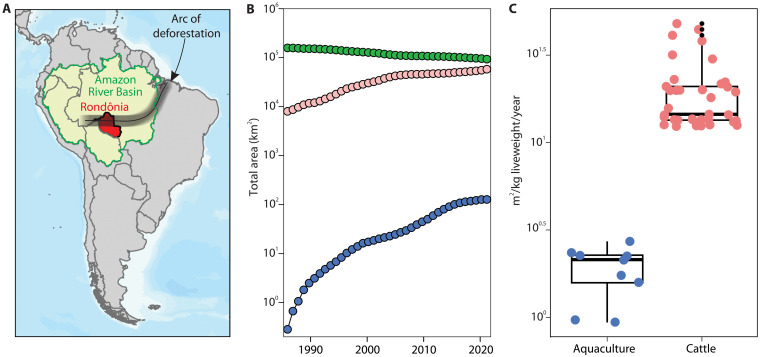
Aquaculture has expanded rapidly in Rondônia in the Brazilian Amazon using land more efficiently than cattle. (**A**) Location of Rondônia in the arc of deforestation. (**B**) Cumulative area of standing forest (green circles) and land converted for aquaculture (blue circles) and cattle farming (pink circles) from 1985 to 2022 in Rondônia. (**C**) Farm-level land-use efficiency measured as square meters needed to produce a kilogram of live weight per year for aquaculture (blue dots) and cattle (pink dots), with box plots indicating median, quartiles, and outliers. This comparison reflects direct on-farm land use only. In Rondônia, aquaculture is predominantly semi-intensive, and the land required to produce feed ingredients can exceed pond area, often by up to threefold, and is typically displaced outside the region, whereas cattle production is largely pasture-fed and directly tied to local land occupation. Data on forest and pasture area in (B) were obtained from the MapBiomas Project–Collection 8 of the Annual Land Use and Land Cover Maps of Brazil, accessed on 10 February 2025 through the link: https://brasil.mapbiomas.org/en/colecoes-mapbiomas/. Data in (C) on fish production were obtained from the Brazilian Institute of Geography and Statistics (IBGE), Table 3940–Aquaculture Production by Product Type, accessed on 05 March 2025, through: https://sidra.ibge.gov.br/tabela/3940. Data in (C) on cattle production were also obtained from IBGE, Table 3939–Livestock Population by Type, accessed on 05 March 2025, through: https://sidra.ibge.gov.br/tabela/3939.

Here, we use AI-enhanced recognition of remotely sensed images from 1985 to 2022 to quantify the land-use trajectory of aquaculture expansion in Rondônia, a state in the Brazilian Amazon comparable in size to that of the United Kingdom (Fig. 1A). Rondônia, located at the heart of the Amazon’s “arc of deforestation,” is the leading producer of native cultured fish in Brazil. Aquaculture production in the region is dominated by semi-intensive freshwater pond systems based on native species, which rely on formulated feeds (*5*). Rondônia has been an epicenter of deforestation in the Amazon’s arc of deforestation, where habitat conversion has been primarily driven by cattle ranching since the 1970s (*6*–*8*). Across this region, extensive clearing through the 1980s, 1990s, and early 2000s converted large areas of forest to pasture, making pasturelands one of the dominant postdeforestation land uses (*6*, *9*–*11*). Much of the remaining pasture is now degraded because of unsustainable land practices, and pasture-driven deforestation continues to push the agricultural frontier further into pristine forested areas (*12*). Alongside the well-documented expansion of pasture areas, we found that the land footprint of aquaculture increased ∼450-fold between 1985 and 2022 (Fig. 1B and table S1) and aquaculture ponds now account for ∼5% (127 km^2^) of the state’s water surface. However, aquaculture remains far more land-efficient than cattle. Producing 1 kg of live weight required an average of ∼13 m^2^ of pasture for cattle, compared to ∼2 m^2^ of pond area for aquaculture in Rondônia. Over the period analyzed, this corresponded to a direct on-farm land-use requirement approximately 3 to 10 times lower for aquaculture than for cattle ranching, depending on year. This comparison reflects direct on-farm land use only and therefore captures differences in local land occupation intensity rather than full life-cycle land requirements. The land required to produce ingredients for fish feed can exceed pond area, often by up to threefold (*13*). Although feed is manufactured locally, the crops used as ingredients are largely cultivated outside the Amazon region, whereas cattle production is predominantly pasture-fed and directly tied to local land occupation (Fig. 1C and table S2).

## RESULTS AND DISCUSSION

**Fig. 2.**
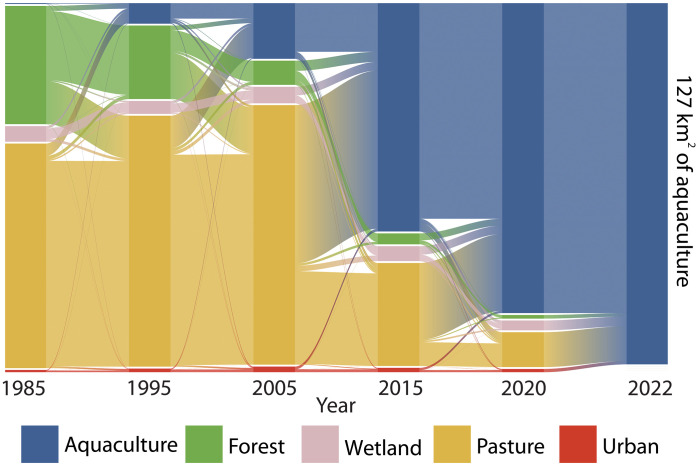
Strategic land-use choices shape the sustainability of aquaculture expansion in the Amazon. Historical land-use classes converted for aquaculture in Rondônia from 1985 to 2022, highlighting transitions from forests, pastures, and wetlands to aquaculture ponds, which now cover 127 km^2^.

We found that aquaculture pond expansion occurred primarily on previously deforested lands (Fig. 2 and data S1), with most ponds built on former pasturelands (87%) and a smaller portion on urban areas or croplands (4%). The remaining aquaculture expansion occurred on natural forests (5%) and wetlands (4%). These land-use transitions show that aquaculture expansion in Rondônia has predominantly occurred on already-cleared lands, particularly former pastures, rather than driving new deforestation fronts. However, the conversion of wetlands or natural forests could have an outsized impact on biodiversity as these areas harbor much greater numbers of species than pastures.

**Fig. 3.**
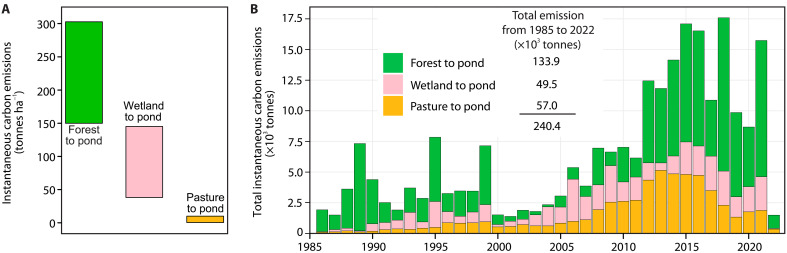
Land-use transitions for aquaculture expansion drive variable carbon emissions in the Amazon. (**A**) Instantaneous carbon emissions per hectare associated with land conversion to aquaculture ponds, showing substantially higher emissions when forests are converted to ponds compared to wetlands or pastures. (**B**) Total carbon emissions from land-use transitions for aquaculture in Rondônia from 1985 to 2022, revealing a peak in emissions from 2010 to 2020, primarily driven by forest and wetland conversion.

Beyond habitat conversion, aquaculture development can also carry substantial carbon costs. For example, accounting for only direct land use of aquaculture, siting ponds on natural forests produces 1.1 to 8.0 times more instantaneous carbon emissions per unit of area from biomass and soil carbon loss than siting on wetlands and 15 to 925 times more emissions than siting on pastures (Fig. 3A). Scaling these per-area estimates to account for the total area under different land uses converted for aquaculture production reveals that the sector’s overall carbon footprint increased markedly between 2010 and 2020, when the amount of forested land converted to aquaculture peaked. This large carbon footprint was evident although the forest area converted to aquaculture was minor compared to pasture-to-pond transitions, which reflects the disproportionate carbon consequences of siting ponds on natural forests (Fig. 3B and table S3).

**Fig. 4.**
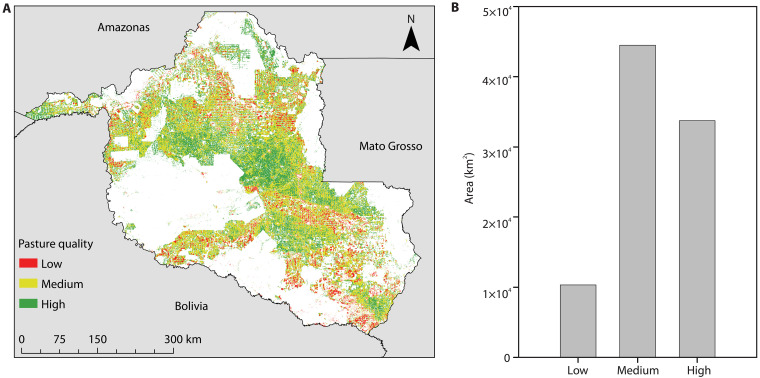
The abundance of degraded pastures offers opportunities for aquaculture expansion. (**A**) Map of pasture quality in Rondônia in 2022, categorized as low (red), medium (yellow), and high (green), showing extensive areas of degraded (low-quality) pastures that could support aquaculture development with minimal environmental trade-offs. (**B**) Total area of pastures by quality, indicating that low-quality pastures cover approximately 10,000 km^2^, while medium- and high-quality pastures cover substantially larger areas. The substantial availability of low-quality pastureland suggests that aquaculture production in Rondônia could potentially be doubled by using just 1.2% of the existing degraded pasture, offering a low-carbon and habitat-friendly pathway for expanding food production in the region. Data from MapBiomas Project—Collection 8 of the Annual Land Use and Land Cover Maps of Brazil, accessed on 10 February 2025 through the link: https://brasil.mapbiomas.org/en/colecoes-mapbiomas/.

Our findings highlight an opportunity to expand animal production in the Amazon by strategically siting aquaculture ponds on already-cleared lands, particularly on degraded pastures, which are widely available (Fig. 4A). Almost 10,000 km^2^ of degraded pastures in Rondônia produce cattle with low efficiency (Fig. 4B). An additional 40,000 km^2^ of pasture is of medium quality, and cattle production in these areas is 20 to 50% less efficient than that in high-quality pastures (*14*). Siting ponds on degraded pasturelands could both mitigate further forest or wetland conversion from aquaculture farm expansion in the Amazon and put already degraded habitats to more productive use. A doubling of aquaculture production could be achieved in the state of Rondônia by converting only 1.2% of the total degraded pasture. As most global aquaculture production is slated to occur inland and in tropical regions with large tracts of forest cover [e.g., Africa (*15*), Asia (*16*)], the dynamics revealed herein are transferable and highlight the importance of better accounting for land-use conversion for incentivizing strategic siting of aquaculture.

Although our results highlight the potential to steer aquaculture expansion toward degraded pastures, there is no evidence indicating that aquaculture has replaced cattle production in the Amazon. Nationally, cattle production in Brazil increased over the study period, indicating that aquaculture expansion in Rondônia occurred alongside, rather than in place of, ongoing growth in the cattle sector. Cattle production remains a major driver of land-use change and, supported by powerful domestic and export-oriented markets (*9*–*11*), has contributed to expansion of the agricultural frontier in a biome central to global biodiversity and climate stability, and one widely recognized as approaching critical ecological “tipping points” (*17*). Against this backdrop, the growth of regional, domestic, and international markets for fish may provide opportunities to transition toward lower-impact animal-sourced food production. Such a transition, however, would also depend on the willingness of farmers and consumers to substitute fish for other animal-sourced foods and would face economic, cultural, and institutional constraints, particularly given the entrenched role of cattle production in Brazil’s economy (*11*). We did not conduct a counterfactual analysis of displacement effects and therefore cannot determine how aquaculture interacts with cattle-driven deforestation dynamics or whether it substitutes for, complements, or indirectly reinforces livestock expansion. Our contribution instead lies in documenting the observed land-use transitions associated with aquaculture growth in isolation and quantifying their associated carbon consequences, while showing that substantially increasing current production would require only a small fraction of the existing pasture area currently available in Rondônia.

Situating aquaculture expansion within the broader landscape context requires considering other potential land-use pathways for degraded pastures. Alternative land-use pathways for degraded pastures, such as regeneration, can deliver climate and biodiversity benefits. In addition to providing habitat for biodiversity, secondary forest regeneration on former pasturelands in the Amazon can accumulate carbon at approximately 2.97 tonnes C ha^−1^ year^−1^ (*18*). However, forest recovery on degraded pasture can be slow, heterogeneous, and contingent on local ecological and management conditions (*19*). Moreover, given that substantially increasing aquaculture production would require only a small fraction of the currently degraded pasture area (e.g., ∼1.2% to double production), the foregone carbon sequestration associated with siting aquaculture on degraded land would represent a relatively small share of the total restoration potential across degraded pastures in Rondônia. Plant-based agriculture represents another potential land-use pathway, such as soybean production, which has historically been a major driver of environmental change in the Amazon. Although plant-based diets are typically associated with lower land-use requirements per unit of protein compared to animal-sourced foods (*20*), cultural preferences in the Amazon favor animal protein sources, which replicates many other emerging economies. In addition, in much of the Amazon, soybean production is largely oriented toward national and international commodity markets and plays a limited role in regional food security and livelihoods, whereas inland fisheries and aquaculture supply protein that is consumed locally. Fish also provide a rich source of nutrients that are difficult to obtain with other sources, primarily omega-3 fatty acids and several micronutrients (*21*). As a result, comparing aquaculture and plant crops requires considerations of additional factors beyond land use, including economic and nutritional dimensions of food systems. Together, these considerations underscore that assessing sustainability outcomes across food production pathways requires integrated evaluation across land, water, and food-system processes.

The environmental impacts of aquaculture go beyond the direct land-use change dynamics analyzed here (*20*). Aquaculture’s land impacts are also tied to feed production, much of which is composed of agricultural commodities like soybean, which also requires land. Additional environmental impacts of aquaculture also include wild fish exploitation for feed (*22*), greenhouse gas emissions from ponds, nutrient and organic matter loading to nearby rivers, water withdrawals, and risks of spreading invasive species. These impacts vary widely across species, production systems, and management intensity, reflecting the heterogeneity of Amazonian aquaculture (*23*). While beyond the scope of our analysis, these additional environmental dimensions associated with aquaculture highlight that aquaculture’s potential to contribute to more sustainable food systems in the Amazon will depend on multiple interacting environmental processes beyond direct land conversion alone.

Aquaculture is the world’s largest source of aquatic food (*24*), and the fast-growing market for freshwater fish could steer its expansion along two distinct pathways. Without proper regulation, deforestation aquaculture (i.e., aquaculture that entails the clearing of natural forests) may perpetuate destructive land-use practices through the siting of ponds in high-value forest habitats. Alternatively, existing policy mechanisms could, in principle, incentivize a food sector that favors conservation-centered aquaculture that leverages already converted habitats. For example, Brazil’s Nationally Determined Contribution under the Paris Agreement includes commitments to restore degraded landscapes, creating a potential policy framework within which sustainable financing for developing aquaculture could tie farmer commitments to both constructing ponds on degraded pasture and setting aside areas for restoration (*25*). These policy mechanisms represent opportunities rather than demonstrated outcomes, and their effectiveness would depend on strong enforcement, safeguards against unintended displacement of land-use pressures, such as the expansion of cattle ranching and feed crops into forest frontiers, and coordination across land-use sectors. In addition, tying sustainably sited aquaculture to the carbon market through offsets or credits could open additional revenue opportunities for ranchers seeking alternatives to cattle production while incentivizing food production with more sustainable land-use implications. Without restricting habitat conversion from agricultural activities, aquaculture could inadvertently lead to additional forest loss if cattle ranchers leverage revenue from fish farming to support pasture expansion. When coupled with policies to curb habitat conversion, sustainably sited aquaculture can provide an alternative income strategy that can return degraded lands to productive use, support forest conservation, and provide release valves for land conversion pressures. These regulatory opportunities and financial incentives could contribute to the development of a new win-win model for aquaculture that aligns food systems and conservation outcomes, but only under conditions that explicitly address enforcement of deforestation controls, and cumulative environmental impacts beyond site-level land conversion.

## MATERIALS AND METHODS

### Fish pond identification input layers

We combined information on generic water bodies identified from satellite imagery with a database of validated aquaculture pond locations in the Brazilian state of Rondônia to train a deep learning model to classify fishponds. The locations of generic water bodies were first identified using a random forest classifier trained on Sentinel-1 and Sentinel-2 satellite imagery from 2019 to 2021 covering the Rondônia study region. This approach provided improved water pixel detection over traditional indices such as the normalized difference water index. Fish pond training data were obtained from a database provided by the Brazilian Agricultural Research Corporation containing the locations of more than 11,000 aquaculture ponds in Rondônia.

### Development, training, and evaluation of the deep learning model

To distinguish aquaculture ponds from generic water bodies identified by the random forest classifier, a U-Net architecture was selected for the deep learning model due to its effectiveness in classification tasks. The model was trained on 128 pixel–by–128 pixel tiles derived from satellite imagery, incorporating multispectral and radar data. Temporal information was included through percentile data, where each band of the input image was represented at seven different percentiles (5, 10, 30, 50, 70, 90, and 95), capturing temporal variations in water bodies, such as changes in water levels or color shifts. To enhance the model’s focus on relevant features, masked pooling was used during the final pooling step of U-Net; this pooling step involved averaging only the water pixels identified in the segmentation step and minimizing the influence of surrounding nonwater features. We further enhanced the model’s ability to recognize complex features specific to water bodies by using contrastive pretraining. This technique leverages a large volume of unlabeled multispectral satellite images by generating two augmented views of each water body, ensuring similar representations for the same water body while differentiating between different ones. This approach improved the model’s recognition of specific water body characteristics rather than general landscape features. The model was trained and validated using data from a subset of municipalities in Rondônia, with testing performed on out-of-sample (data not used in the model training) data from other municipalities in the region. The performance of the model was evaluated on the basis of accuracy, precision, recall, and F1 score, which assess different aspects of classification performance, including overall correctness (accuracy), the proportion of correctly identified positive cases (precision), the ability to capture all relevant instances (recall), and a balanced measure of precision and recall (F1 score). Our model demonstrated superior generalizability to regions not represented in the training data compared to traditional random forest classification methods (Table 1). More details of the model development and performance can be found in Greenstreet *et al.* (*26*).

**Table 1.** Performance of selected models when trained on municipalities in Rondônia and evaluated on unseen municipalities in the Amazon region. Results are shown for a Random Forest (RF) baseline and three U-Net–based approaches (percentile, masked pool, and contrastive). For each model, performance metrics (precision, recall, F1 score, and accuracy) were computed across three independent spatial train–test splits to assess geographic generalization, with each split repeated using five different random seeds to account for stochasticity in model training. Values report the mean performance across all runs, with the accompanying standard deviation quantifying variability across spatial splits and random initializations. See Greenstreet *et al.* (*26*) for full details on data preprocessing, spatial splitting strategy, and model training.

		U-Net models		
Metric	RF	Percentile	Masked pool	Contrastive
Precision	0.34 ± 0.32	0.99 ± 0.01	0.88 ± 0.05	0.94 ± 0.00
Recall	0.40 ± 0.34	0.56 ± 0.04	0.87 ± 0.03	0.85 ± 0.01
F1	0.35 ± 0.30	0.69 ± 0.04	0.87 ± 0.02	0.89 ± 0.00
Accuracy	0.30 ± 0.29	0.78 ± 0.02	0.86 ± 0.03	0.89 ± 0.00

### Land-use trajectory of aquaculture

To understand the land-use transitions associated with aquaculture expansion, we used the identified aquaculture pond locations from our deep learning model. Then, we determined the land cover category of each pond at the beginning of the time series of the MapBiomas (https://mapbiomas.org) database (i.e., 1985) and tracked each pond through time to identify when the area of the pond was converted from nonaquaculture to aquaculture. MapBiomas is a collaborative initiative that uses satellite imagery and machine learning to produce annual land cover and land-use maps, providing detailed historical data on landscape transformations across Brazil.

### Pasture quality assessment

In addition to land-use transitions, we analyzed pasture quality using MapBiomas data, which classifies pastures into three categories—high, medium, and low quality. In terms of environmental quality, these classifications reflect the degradation status and productivity of pastures. High-quality pastures are well managed and have dense vegetation cover, high forage availability, and minimal soil degradation. Medium-quality pastures exhibit signs of degradation, such as reduced vegetation cover, soil compaction, and declining productivity. Low-quality pastures are heavily degraded and often characterized by exposed soil, invasive species, reduced biodiversity, and poor forage conditions, making them less suitable for livestock grazing and more prone to conversion into other land uses, such as aquaculture.

### Carbon footprint associated with land-use change

The carbon footprint associated with aquaculture-driven land-use change was estimated by using the instantaneous carbon emissions attributable to converting nonaquaculture habitat to aquaculture ponds. These emission factors were obtained from the Intergovernmental Panel on Climate Change and the Brazilian inventory and encompass the carbon stocks and sequestration potential of each land-use category—forest, pasture, and wetland (*27*). Last, we paired these estimates of carbon emissions per hectare with the amount of area converted from nonaquaculture to aquaculture uses to obtain yearly estimates of carbon emissions associated with aquaculture expansion.
