# Supplementary material for: Land-use transitions and carbon emissions of aquaculture expansion in the Brazilian Amazon
Source: Sci Adv. 2026 Aug 14;12(33):eaec5472. doi: 10.1126/sciadv.aec5472 (PMC13475470; doi:10.1126/sciadv.aec5472)
Supplement: Supplementary file 1 [file sciadv.aec5472_sm.pdf]

Science Advances
AAAS

Supplementary Materials for

Land-use transitions and carbon emissions of aquaculture expansion in the Brazilian Amazon

Felipe S. Pacheco *et al.*

Corresponding author: Felipe S. Pacheco, felipe.pacheco@cornell.edu; Carla P. Gomes, gomes@cs.cornell.edu; Alexander S. Flecker, asf3@cornell.edu

Sci. Adv. **12**, eaec5472 (2026)
DOI: 10.1126/sciadv.aec5472

The PDF file includes:

Tables S1 to S3
Legend for data S1

Other Supplementary Material for this manuscript includes the following:

Data S1

Table S1.

Annual extent of aquaculture, pasture, and forest in Rondônia (1985–2022), reported in km². Data on forest and pasture area were obtained from the MapBiomas Project Collection 8 of the Annual Land Use and Land Cover Maps of Brazil, accessed on February 10, 2025 (https://brasil.mapbiomas.org/en/colecoes-mapbiomas/), and aquaculture area data were derived from this study (see Data S1 in the Supplementary Materials).

Year	Area (km^2)			Year	Area (km^2)		
	Aquaculture	Pasture	Forest		Aquaculture	Pasture	Forest
1985	0.28	16484.3	208122	2004	22.72	64909.3	159315
1986	0.66	17663.1	206944	2005	24.59	68082.6	155929
1987	1.06	19437.5	205154	2006	27.07	69990.3	153850
1988	1.80	21422.4	203174	2007	29.57	71389.3	152328
1989	2.48	22767	201665	2008	33.76	72305	151334
1990	3.10	23271	201078	2009	39.10	72585.9	150901
1991	3.90	24780.2	199480	2010	44.56	72734.8	150635
1992	4.78	27057.4	197172	2011	50.22	73685.6	149629
1993	5.70	29512.1	194741	2012	59.42	74308.4	148827
1994	6.79	32699.1	191625	2013	69.85	74844.2	148222
1995	8.27	36768.6	187579	2014	80.27	75293.2	147321
1996	10.23	40687.1	183702	2015	90.52	76209	145909
1997	12.12	43299	181117	2016	100.97	77756.8	144190
1998	14.00	45665.9	178772	2017	108.73	79024.1	142568
1999	16.27	48581.4	175891	2018	114.47	80022.5	141222
2000	17.37	51117.1	173540	2019	117.67	81736.5	139284
2001	18.54	53755.4	170890	2020	121.77	83181.7	137679
2002	20.03	56917	167658	2021	126.63	85373.2	135488
2003	21.35	61350.3	163054	2022	127.38	88851.5	132947

Table S2.

Farm-level land-use efficiency measured as m^2 needed to produce a kilogram of live weight per year for aquaculture and cattle. Data of fish production from the Brazilian Institute of Geography and Statistics, Table 3940 - Aquaculture Production, by Product Type, accessed on March 5 2025 through the link: https://sidra.ibge.gov.br/tabela/3940. Data of cattle production from the Brazilian Institute of Geography and Statistics, Table 3939 - Livestock population by type, accessed on March 5 2025 through the link https://sidra.ibge.gov.br/Tabela/3939.

m^2 / kg liveweight / yr		
aquaculture	cattle	
2.35	47.93	13.52
0.93	44.39	13.33
0.97	41.10	13.54
1.58	38.15	14.41
1.74	31.74	14.38
2.14	30.09	13.99
2.23	19.48	13.65
2.27	21.68	13.44
2.72	19.96	13.51
	20.94	13.49
	20.80	13.13
	22.96	12.64
	22.22	12.63
	19.88	12.46
	19.84	12.38
	20.05	12.66
	18.09	12.49
	15.73	12.56
	14.52	

Table S3.

Annual carbon emissions from land-use transitions to aquaculture in Rondônia (1985-2022). Emissions are shown by previous land cover in tons of carbon per year.

	tons of carbon		
Year	Forest	Wetland	Grassland
1985	1112.87	99.19	46.13
1986	1779.20	45.72	100.89
1987	1187.69	184.21	130.24
1988	3195.49	216.40	206.95
1989	7117.48	84.81	125.87
1990	3603.04	605.67	183.04
1991	1623.98	583.45	307.67
1992	829.17	715.03	367.75
1993	1995.58	1377.03	335.54
1994	1923.85	513.64	423.70
1995	5242.38	2107.22	493.96
1996	1477.58	905.85	871.37
1997	2064.99	560.80	837.03
1998	1707.52	879.66	863.77
1999	4806.53	1391.93	951.01
2000	786.27	195.90	528.70
2001	388.69	431.11	567.21
2002	732.48	440.41	717.41
2003	275.98	896.72	618.38
2004	177.43	1550.67	615.10
2005	903.76	1311.52	837.22
2006	948.45	3438.19	986.38
2007	857.59	1893.67	1114.17
2008	2999.60	2022.17	1937.20
2009	1117.11	2976.14	2547.95
2010	2830.83	1576.94	2619.09
2011	1559.04	1888.61	2706.40
2012	6688.93	1406.64	4348.56
2013	6075.81	603.87	5143.86
2014	7830.99	1455.28	4857.39
2015	9632.23	2645.43	4811.93
2016	9391.11	2393.31	4736.01
2017	4570.11	2802.32	3497.47
2018	12508.14	2783.55	2298.30
2019	6871.68	1655.68	1331.21
2020	4870.33	2047.21	1759.45
2021	11114.40	2741.85	1878.69
2022	1085.72	53.81	351.81

Data S1. (separate file)

Geospatial dataset of aquaculture ponds and associated land-use transitions in Rondônia, Brazil. Each row represents an individual aquaculture pond identified from satellite imagery. The columns include: ID, unique identifier for each pond; long and lat, geographic coordinates of the pond centroid in decimal degrees (WGS84); Area_km², surface area of the aquaculture pond in square kilometers; Year of construction, estimated year when the pond was converted to aquaculture; Use before construction, dominant land cover prior to aquaculture establishment; Emission (ton Carbon), estimated carbon emissions associated with land-use conversion to aquaculture, expressed in tons of carbon (t C); Y1985, Y1995, Y2005, Y2015, Y2020, and Y2023, land-use classification at the pond location for the corresponding year, indicating the dominant land cover category (e.g., Forest, Grassland, Wetland, or Aquaculture).
